# Assessing the morphology and bone mineral density of the immature pars lateralis as an indicator of age

**DOI:** 10.1007/s00414-023-03085-z

**Published:** 2023-09-29

**Authors:** Roxanne Thornton, Mira G. Mendelow, Erin F. Hutchinson

**Affiliations:** School of Anatomical Sciences, Faculty of Health Sciences, University of the Witwatersrand, Johannesburg, South Africa

**Keywords:** Pars lateralis, Fetal osteology, Geometric morphometrics, Bone mineral density, Bone morphology, Forensic identification

## Abstract

Age estimation is crucial when the state of personhood is a mitigating factor in the identification of immature human remains. The maturation sequence of immature bones is a valuable alternative to dental development and eruption standards. Bordering the foramen magnum and pars basilaris, the pars lateralis is somewhat understudied. The aim of this study was to comprehensively describe the morphology of the immature human pars lateralis bone. Human pars laterali were sourced from the crania of 103 immature individuals of unknown provenance from the Johannesburg Forensic Paediatric Collection (JFPC), University of the Witwatersrand (HREC-Medical: M210855). The study sample was subdivided into early prenatal (younger than 30 gestational weeks; *n* = 32), prenatal (30–40 gestational weeks, *n* = 41) and postnatal (birth to 7.5 months, *n* = 30) age groups. The morphology of the pars laterali was studied using a combination of bone mineral density pattern assessments, geometric morphometrics and stereomicroscopy. Bone mineral density in postnatal individuals was lower when compared with the prenatal individuals. No statistically significant differences between density points were noted. The overall shape of the pars lateralis changed from a triangular shape in the early prenatal individuals to a fan-like quadrilateral bone in postnatal individuals. The angulation of the medial border for the foramen magnum highlighted a change in shape between straight in the early prenatal cohort to V-shaped in the postnatal individuals. The various technical approaches used in the current study provided detailed descriptions of the pars lateralis which establishes a valuable foundation for diagnostic criteria employing morphological predictors for biological profiling.

## Introduction

In the establishment of a biological profile within the forensic, archaeological, and legal contexts, age estimation is a fundamental and often critical requirement [1–3]. Age estimation is pertinent particularly when identifying immature human remains in cases of criminal activity such as suspected infanticide, where the state of personhood or separate existence is a mitigating factor [4–6]. Whilst dental development and eruption sequences are regarded as the gold standard in age estimations of immature human remains [7, 8], often alternative methods investigating the morphology of the skeleton are required when the dentition is absent. Thus, a wide range of method options is deemed highly desirable, particularly in cases where skeletal elements may be recovered in isolation or in a damaged state [3].

The maturation sequence of bones related to the immature human skeleton serves as a valuable alternative to dental development and eruption standards and various elements related to both the cranial and postcranial skeleton have been investigated [9, 10]. The basicranium serves as a focal point for growth-related investigations, owing to its association with the development and growth of the brain and related soft tissue structures and is derived from 25 individual ossification centres, which at birth are localised to predominantly the occipital and sphenoid bones [1, 10]. The development and growth of the occipital bone are of particular interest given its contributions to the borders of the foramen magnum as well as early maturation relative to other basicranial elements [1, 11–13].

Located within the posterior cranial fossa, the immature occipital bone consists of the pars squama, pars laterali and the pars basilaris [14], all of which come together to form a single adult derivative. Whilst the development and growth of the pars basilaris has been well documented relative to cranial base flexion and elongation [15–23], a paucity exists in the literature describing the osteology of the pars lateralis.

**Fig. 1:**
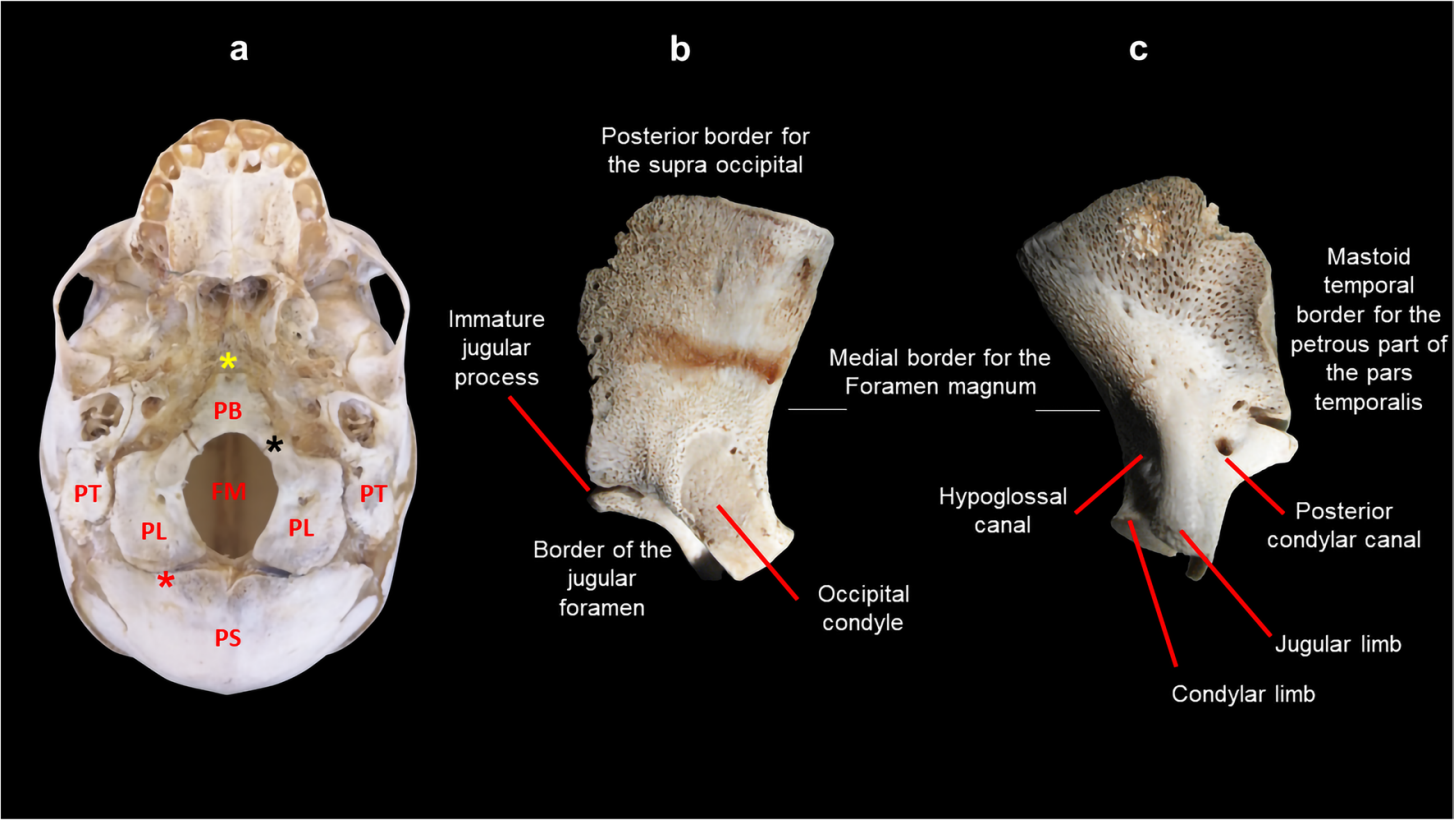
Prenatal basicranium representing osteological features and borders of the left pars lateralis. **a** Extracranial view of the basicranium highlighting the PS: pars squama, PL: pars laterali, FM: foramen magnum, PB: pars basilaris, PT: pars temporalis and synchondroses* (red: PIOS—posterior intraoccipital synchondrosis, black: AIOS—anterior intraoccipital synchondrosis and yellow: SOS—sphenoid-occipital synchondrosis). **b** Extracranial view of the left pars lateralis representing posterior border for the supraoccipital, immature jugular process, border of the jugular foramen, occipital condyle and medial border for the foramen magnum. **c** Intracranial view of the left pars lateralis representing mastoid temporal border for the petrous part of the pars temporalis, posterior condylar canal, jugular and condylar limb contributing the hypoglossal canal and medial border for the foramen magnum. Magnification (1.5–2.5 ×) performed on Nikon Stereomicroscope (SMZ1500, Japan)

The pars lateralis in conjunction with the pars squama of the immature occipital bone commences with endochondral ossification as early as the eighth week of gestation [10]. In the immature cranial base, the pars lateralis is bordered anterolaterally by the petromastoid components of the immature temporal bone and superiorly by the parietal bone [10]. In addition, the pars lateralis shares the posterior intraoccipital synchondrosis (PIOS) with the pars squama and the anterior intraoccipital synchondroses (AIOS) with the pars basilaris [11] (Fig. 1). Constituting the lateral border of the foramen magnum, the pars lateralis also provides a platform upon which the cerebellum of the brain rests as well as a point of transition for the hypoglossal nerve through the hypoglossal canal in the posterior cranial fossa. Whilst located in a relatively stable area of the cranial base, which has a higher probability of recovery and is relatively resistant to postmortem damage, the pars lateralis is somewhat understudied.

Fazekas and Kosa [16], Thornton et al. [2] and recently Figueiro et al. [3] have documented changes in the size parameters of the pars lateralis across various samples. Fazekas and Kosa [16] assessed the length and width of the pars lateralis in a Hungarian sample aged between 12 gestational weeks and birth, noting increases in the general dimensions of this bone with age [16]. Thornton et al. [2] in a South African sample expanded on the previously documented prenatal age range to also assess the length and width of the pars lateralis in postnatal (birth to 7.5 months) individuals. In a sample of 74 modern humans, significant increases in the length and width of the pars lateralis were observed across all age groups [2]. Figueiro et al. [3] in a sample of 127 individuals aged between 6 months prenatal and 4 years postnatal expanded on the traditional length and width measurements to further include investigations of length and width of the anterior synchondrosis and occipital condyle, respectively, with the explicit aim of using regression functions for each of the assessed measurements. In their assessment of the pars lateralis, Figueiro et al. [3] noted that functions derived from all measurements were useful across the period of 6 months prenatal to 2 years postnatal and are applicable to incomplete pars laterali, which is particularly useful across a wide range of contexts [3]. Whilst illustrating changes in the dimensions of the pars lateralis across various age intervals, these metric methods are somewhat limited in their usefulness to unfused basicranial or foetal materials [1].

Investigations of the morphology of the pars lateralis are particularly important within a forensic setting, given the perinatal version of this bone when viewed from the intracranial surface bears a close resemblance to the dorsal surface of the scapula [10]. Redfield [15] described a series of age-related changes across the occipital bone in an archaeological sample, providing limited detailed descriptions to the hypoglossal canal and the two occipital synchondroses at the anterior and posterior ends of the pars lateralis [15]. Subsequent assessments of the morphology of the pars lateralis have recently been limited to studies of the fusion times of the PIOS and AIOS, respectively [1, 11]. Coqueugniot and Le Minor [11] in a sample of 152 skulls categorised the maturation of the SIOA and SIOP as well as the shape of the foramen magnum. Whilst no differences across the sexes were observed in terms of fusion times across the PIOS or AIOS, a high correlation between the shape of the foramen magnum and chronological age was noted up to the point of synchondrosis closure [11]. Cardoso et al. [1] in a sample of 64 individuals aged between birth and 8 years also documented the stages of fusion between the condylar and jugular limbs which contribute to the formation of the hypoglossal canal. The PIOS fused earlier than the AIOS. Thus far morphological studies have been localised to the two synchondroses with some focus on the hypoglossal canal [1, 11].

Whilst documenting changes in the fusion of the PIOS and AIOS, potential changes in the morphology as influenced by increases in the size of the bone have largely been overlooked. Furthermore, few studies have paid attention to changes in the morphology of the pars lateralis during the transition between the prenatal and postnatal stages of growth, which are important when considering changes in the biomechanical stresses imposed on the cranial based from the neck region [12, 13, 24]. Whilst some changes may be the result of general increases in size-associated growth, others may be the result of biomechanical influences. Assessments of bone density changes across various elements of the immature skeleton including the mandible [25] and ear ossicles [26] have proven useful in showing the potential effects of biomechanical influences on changes in the morphology of bones with growth. Hutchinson et al. [25] in a sample of 45 mandibles aged between 30 gestational weeks and 5 years of age provided insights into the direction of growth of the mandible relative to dental eruption [25]. Morris et al. [26] showing a pattern of change in bone density relative to function across the ear ossicles illustrated how bone density patterns could be used to reflect changes in the functional environment [26]. These relationships between changes in bone mineral density patterns and growth of immature skeletal elements have provided valuable insights into how the morphology of the bone is influenced with age. Thus, the influence of the interplay between size and shape is also of importance when considering changes in the morphology of a bone with age within a forensic setting.

Whilst traditional morphometric techniques have been of value, they are largely restricted to two-dimensional analyses and as such the use of three-dimensional analyses such as geometric morphometrics is recommended. As such the aim of this study is to describe changes in the morphology of the pars lateralis, considering the interplay between size and shape using geometric morphometrics as well as stereomicroscopy and bone density analyses.

## Materials and methods

### Sample

Human skeletal elements (pars laterali) were sourced from the crania of 103 foetal and infant individuals of unknown provenance from the Johannesburg Forensic Paediatric Collection (JFPC), Department of Forensic Medicine and Pathology, University of the Witwatersrand. The JFPC is an ongoing humanitarian and scientific initiative, which aims to preserve and safeguard unidentified immature skeletal remains. Individuals included in the JFPC are unclaimed and unidentified decedents previously admitted to the Forensic Pathology Services for medicolegal examination. Ethical approval was granted through the Human Research Ethics Committee of the University of the Witwatersrand (HREC-Medical: M210855).

In South Africa, prenatal and postnatal remains are admitted to the forensic pathology services in accordance with legislature regulating forensic services [4–6]. Legal viability is established when a foetus has experienced at least 26 gestational weeks intrauterine life before expulsion. As such, individuals aged less than 30 gestational weeks represent early prenatal category which is associated with nonviable decedents ascertained with autopsy as illegal abortion cases. The prenatal category (30–40 gestational weeks) represents viable individuals which were admitted to the forensic pathology services under the circumstances of death which include illegal abortion, concealment of birth and stillborn decedents. The postnatal age category represents individuals admitted to the forensic pathology services as suspected infanticide cases. Individuals admitted to the Forensic Pathology services are thus processed to firstly ascertain age and then determine the circumstances of potential infanticide.

Individuals were previously aged using dental radiographs taken with a Nomad portable X-ray scanner (Aribex, Charlotte, NC, USA), and age was assigned based on dental ageing criteria [7]. Those individuals presenting with missing or damaged dentition were aged using anthropometric standards of femoral elements [27]. Subsequent to biological age estimations and based on the above criteria, the study sample was subdivided into early prenatal (younger than 30 gestational weeks, *n* = 32), prenatal (30–40 gestational weeks, *n* = 41) and postnatal (birth to 7.5 months, *n* = 30) age groups (Table 1). Biological sex was assigned at autopsy or via molecular assay depending on the condition of remains [28] (Table 1). Individuals were excluded from analyses in the event of obvious developmental abnormalities or in cases where postmortem damage was evident on bone surfaces, regions of interest or sites of landmark plotting. The right pars lateralis was used for individuals where the left pars lateralis was excluded. Thus, sample sizes for each technique varied based on the above criteria.

**Table 1:** Age and sex distribution of the sample applied to methods incorporated in the study

Dental and anthropometric age estimates	Biological sex*		Method		
Age categories	Female	Male	Bone mineral density (BMD)	Geometric morphometrics	Stereomicroscopy
Early prenatal (< 30 gestational weeks)	13	11	19	32	30
Prenatal (30–40 gestational weeks)	12	13	20	39	41
Postnatal (birth–7.5 months)	20	9	22	30	30
Total (*N*)	45	33	61	101	101

^*^Biological sex determined at autopsy (when possible) or via molecular assay (Thornton et al., 2021)

## Methods

### Bone mineral density patterning

Bone mineral density (BMD) is defined as the measure of inorganic mineral content present in bone [29], and thus the degree of mineral content present is indicative of bone quality. To assess bone mineral density across individuals within the sample, the pars laterali elements were scanned using a Nikon XTH 225L micro-focus CT X-ray unit (Nikon Metrology, Leuven, Belgium) located at the MIXRAD facility of the South African Nuclear Energy Corporation (NECSA).

Scanning parameters were set to 100 kV/100 µA and 100 µm as a means of standardising the scanning conditions across the sample. A 0.1-mm thick aluminium filter was used to approximate a homogeneous X-ray beam spectrum by removing the lower energy photons. Individual bones were securely mounted in OASIS floral foam (OASIS Floral Products, Gauteng, South Africa) and orientated in the intracranial anatomical perspective. A material of uniform composition was used as a density reference value across all scans. The mounted bones were then placed on to a rotating sample manipulator, which facilitated scanning at 360° and one-thousand projection images were obtained resulting in a good-quality scan. The scanning setup was optimised for the highest spatial resolution where a resolution of between 0.023 and 0.050 µm was obtained. Subsequent to scanning, volume files were then reconstructed using NIKON CTPRO software (Nikon Metrology, Leuven, Belgium). After three-dimensional reconstruction, all the volume files were imported into VGStudio Max V2.2 (Volume Graphics GmbH, Heidelberg, Germany) for further analysis.

**Fig. 2:**
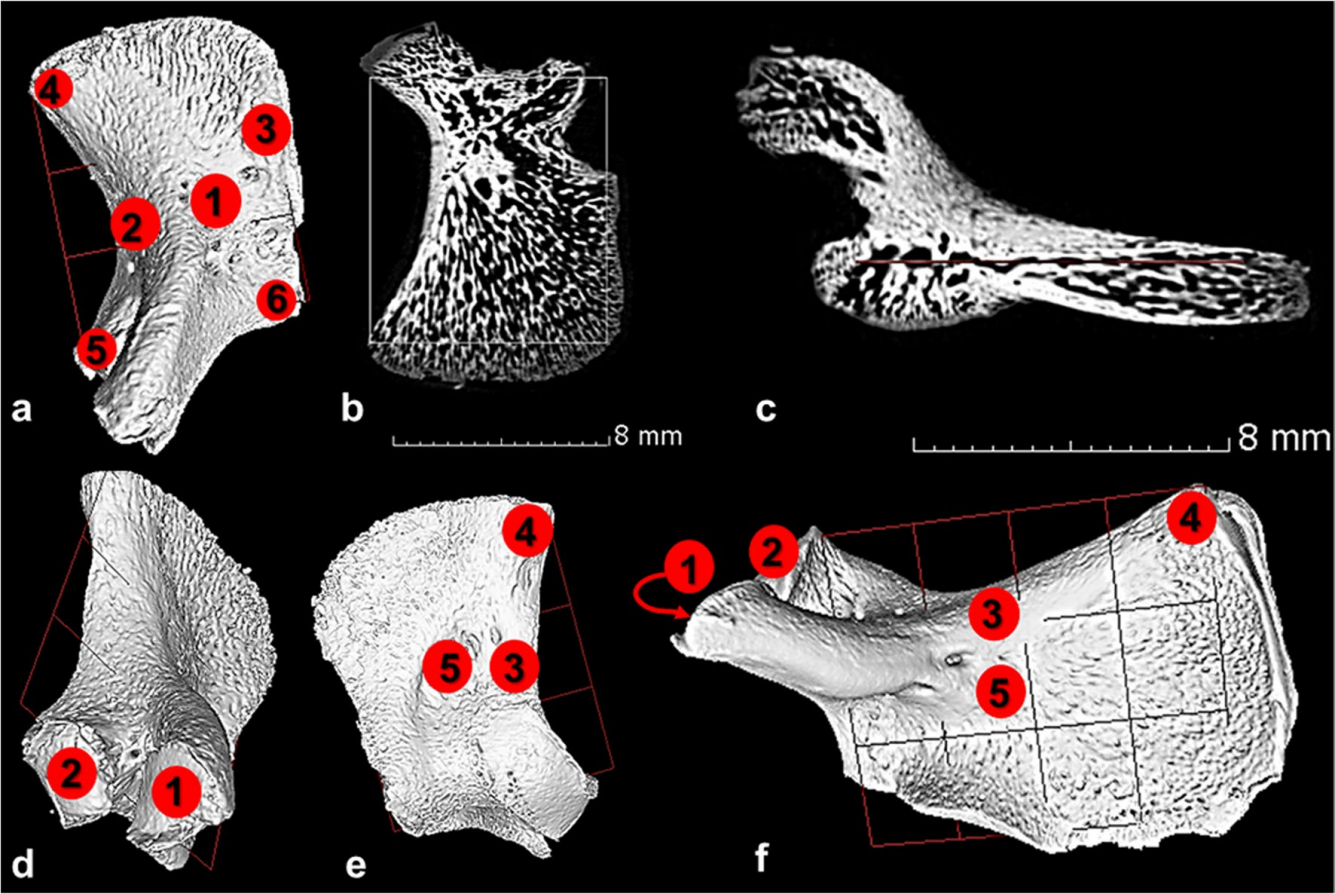
Reference planes and regions of interest for the pars lateralis bone. Reference planes (red grid) for the pars lateralis bone. **a** 3D reconstruction of the early prenatal left pars lateralis with reference points for reference plane generation: primary ossification site (1), the midline point on inferior border for the foramen magnum (2), medial edge of the posterior region for the supraoccipital (pars squama) (3), the midline point on medial posterior border (4), the posterior border of the condylar limb (5) and immature jugular process (6). **b** Transverse section of the early prenatal left pars lateralis: white border represents reference plane. **c** Coronal section of the early prenatal left pars lateralis. **d** Inferior view of the early prenatal left pars lateralis bone. **e** Extracranial view of the early prenatal left pars lateralis bone. **f** Intracranial view of the postnatal left pars lateralis bone. Regions of interest: (1) jugular limb facet, (2) condylar limb facet, (3) inferior border of the foramen magnum, (4) medial edge of the posterior region for the supraoccipital (pars squama) of the occipital complex, (5) primary ossification site. Images generated from VGStudio Max V2.2 software

For clear estimation of surface landmarks, all imported volume files were subjected to a surface estimation analysis to allow for the selection of landmarks digitally. The orientation of the Micro-CT slices of the pars laterali elements was then standardised by aligning each slice to a predetermined transverse reference plane. The transverse reference plane for each bone was established by selecting a minimum of three reference points across the x, y, and z planes, along the intracranial, extracranial, and lateral view of the pars lateralis. Reference points included the primary ossification site (1), the midline point on inferior border for the foramen magnum (2), medial edge of the posterior region for the supraoccipital (pars squama) (3), the midline point on mastoid posterior border (4), the posterior border of the condylar limb (5) and immature jugular process (6) (Fig. 2a–c). Once the transverse reference plane was established, Micro-CT slices were aligned to this plane and regions of interest were selected and analysed across all surfaces of the pars lateralis to ascertain the bone density distribution. Each region of interest included a radius of 1.5 mm and thickness of 1.0 mm to assess bone density distribution across a series of slices. Regions of interest included the jugular limb facet, condylar limb facet, inferior border of the foramen magnum, medial edge of the posterior region for the supraoccipital (pars squama) of the occipital complex and primary ossification site (Fig. 2d–f).

### Geometric morphometrics

**Fig. 3:**
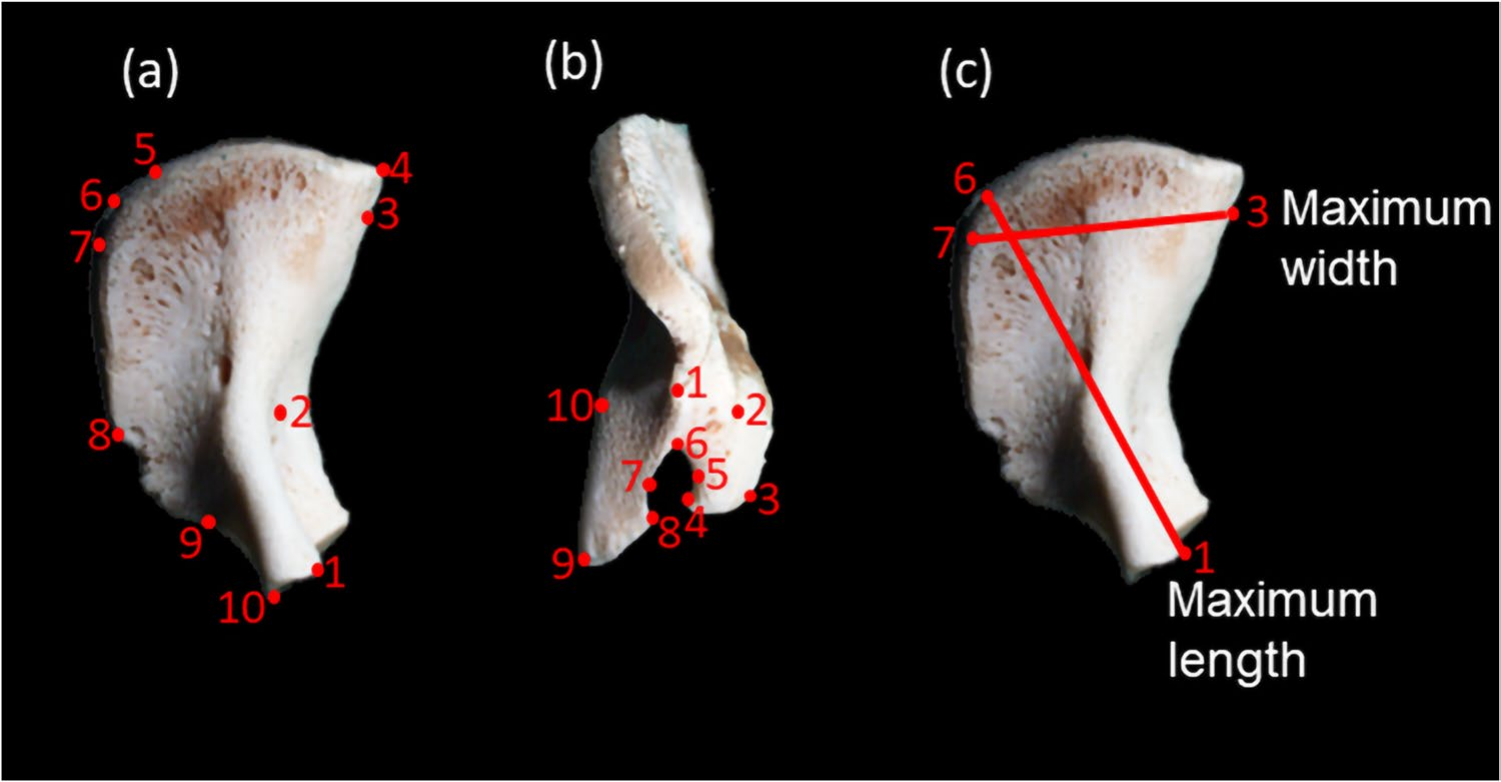
Pars lateralis indicating the position of fixed and floating landmarks and measurements as adapted from Schaefer et al. (2009) and Zdilla et al. (2021). **a** Intracranial view of prenatal pars lateralis. Fixed landmarks: (1) superior jugular limb, (2) anteromedial angle, (4) posteromedial corner, (7 and 8) lateral facet, (9) anterolateral corner, (10) inferior jugular limb. Floating landmarks: (3) medial border, (5) superior posterior border, (6) mid posterior border. **b** Hypoglossal region of prenatal pars lateralis. Fixed landmarks: (1) posterior corner, (2) jugular tubercle, (3 and 4) jugular limb, (6) anteromedial angle, (8 and 9) condylar limb. Floating landmarks: (5) jugular mid anteromedial angle, (7) condylar mid anteromedial angle. **c** Measurements to assess pars lateralis size. Maximum length: the greatest distance between the anterior and posterior interoccipital synchondrosis. Maximum width: the greatest distance between the medial and lateral margins of the posterior interoccipital synchondrosis

To assess size and shape changes of the pars lateralis relative to each age group, a Microscribe® G2 digitiser (Immersion Corp., San Jose, California, USA) was used to digitise a series of fixed and floating landmarks. The general outline of the pars lateralis was first digitised to study the overall shape of the bone relative to each age group. Each pars lateralis was positioned on Affinis Perfect impressions morphometric putty (Coltene Holding Co., Altstatten, Switzerland) with the jugular limb facing towards the viewer, producing an intracranial view of the bone (Fig. 3a). A total of 10 landmarks were digitised, of which seven were fixed and three were floating (Table 2). The pars lateralis was then reorientated (Fig. 3b), to allow for digitising of the hypoglossal region. The posterior border of the bone was cushioned on OASIS floral foam (OASIS Floral Products, Gauteng, South Africa) with the lateral aspect facing the viewer. A total of 10 landmarks were digitised along the perimeter of the tubercles of the condylar and jugular limbs, of which eight were fixed and two were floating (Table 2).

**Table 2:** Landmarks digitised on the pars lateralis

Number	Osteological name	Description (adapted from Schaefer et al., 2009)
Pars lateralis		
1*	Superior jugular limb	The furthest point on the jugular limb (superiorly)
2*	Anteromedial angle	Angle between projections of the jugular and condylar limb
3	Medial border for foramen magnum	Beginning point of curvature on posterior aspect below posteromedial corner
4*	Posteromedial corner	Point where the medial and posterior borders intersect
5	Superior posterior border	Midpoint on posterior border, articulates with supraoccipital
6	Mid posterior border	The most lateral point on curvature of the posterior border
7*	Posterior lateral facet	The most posterior point of facet on lateral border, articulates with temporal-mastoid
8*	Anterior lateral facet	The most anterior point of facet on lateral border, articulates with temporal-mastoid
9*	Border of jugular foramen	Indentation between the lateral border and jugular limb
10*	Inferior jugular limb	The furthest point on the jugular limb (inferiorly)
Hypoglossal region		
1*	Posterior corner	The most anterior point on posterior border
2*	Jugular tubercle	The furthest point on the jugular limb
3*	Superior jugular limb	Inner tip of the jugular limb
4*	Inferior jugular limb	Outer tip of the jugular limb
5	Jugular mid anteromedial angle	Midway way between the jugular deep anteromedial angle and the superior jugular limb
6*	Anteromedial angle	Angle between projections of the jugular and condylar limbs
7	Condylar mid anteromedial angle	Midway way between the condylar deep anteromedial angle and the superior condylar limb
8*	Superior condylar limb	Inner tip of the condylar limb
9*	Inferior condylar limb	Outer tip of the condylar limb
10*	Condylar tubercle	The furthest point on the condylar limb

^*^Fixed landmark

Refer to Fig. 1 for general anatomy and borders of the pars lateralis

The size of the pars lateralis was assessed by measuring the maximum length and maximum width using the digitised landmark data. The maximum length was defined as the greatest distance between the anterior and posterior interoccipital synchondrosis, i.e. between the superior jugular limb (landmark 1) and the mid posterior border (landmark 6). Maximum width was defined as the greatest distance between the medial (superior posterior border; landmark 5) and lateral margins (posterior lateral facet; landmark 7) of the posterior interoccipital synchondrosis (Fig. 3c). Measurements (Fig. 3c) were calculated as the distance between two digitised landmark points using the three-dimensional Pythagorean formula [30].

### Stereomicroscopy

Stereomicroscopy of the intracranial, extracranial, superior, posterior, and lateral orientations of the left pars lateralis was performed using a stereomicroscope (Nikon SMZ1500, Japan). Magnification was applied for clarification of feature development and ranged between 0.75 × and 3.5 × depending on the size of the bone. A digital catalogue of images reflecting the morphology of each surface was recorded. Images were arranged to reflect the progression of changes in the morphology of each skeletal element according to the stage of development. Morphological descriptions were provided to reflect changes in specific features across early gestation (< 30 gestational weeks) and the first year of life. Descriptions expanded on previous morphological summaries complied for identification by Cunningham et al. [10]. The absence or presence of specific features was recorded (Table 3).

**Table 3:** Visualised bone morphology associated with foetal and postnatal age ranges (adapted from Cunningham et al., 2016)

Trait	Description	Recorded data
Bone shape		
Triangular	Represented by thickened medial border with two projecting limbs and rounded posterior/mastoid border	Absent/present
Quadrilateral	Represented by thickened medial border with two projecting limbs and wedge-like posterior/mastoid border	Absent/present
Fan-like quadrilateral	Represented by thickened medial border with two curved projecting limbs and surface extending towards the temporal and pars squama bones	Absent/present
Intracranial surface		
Centre ossification pattern	Radiating from primary ossification site Uniform and advanced ossification represented by cortical smooth bone Differential and delayed ossification represented by trabecular and porous bone Typically, near the mastoid temporal border with nutrient foramina	Uniform/differential
Posterior condylar canal	Variations in location and size adjacent to the jugular limb projection	Absent/present
Border morphology	90° angle of the border for the supraoccipital Serrated appearance of the mastoid temporal border	Absent/present Absent/present
Medial border for the foramen magnum		
Medial border	Straight and thin	Absent/present
	Curved and thickened	Absent/present
	V-shaped	Absent/present
Condylar and jugular articulation facets		
Shape of facet	Square	Absent/present
	Round	Absent/present
	Pointed	Absent/present
Metaphyseal surface	Smooth cortical bone	Absent/present
	Ridgelike metaphyseal surface with lipping of borders	Absent/present
Nutrient foramina		
Presence of singular or multiple	Observed on the intracranial and extracranial surface and intersection of jugular and condylar limbs	Absent/present
Extracranial surface		
Ossification pattern	Radiating from the centre of the bone Uniform ossification represented by no differentiation on surface and cortical bone Differential ossification represented by a combination of trabecular, cortical bone and longitudinal striations at the mastoid temporal border	Uniform/differential
Posterior condylar canal	Variations in location and size adjacent to the occipital condyle	Absent/present
Jugular foramen and process	Rounded border for immature jugular foramen, presence of jugular process	Absent/present
Occipital condyle	Smooth Raised and ridgelike Billowing of superior border	Absent/present Absent/present Absent/present
Hypoglossal canal region		
Jugular limb	Straight and thickened protuberance Curved and thickened protuberance Oval eminence present Hooking of articulation facet	Absent/present Absent/present Absent/present Absent/present
Condylar limb	Straight and thickened protuberance Curved and thickened protuberance Shorter than the jugular limb Hooking of articulation facet	Absent/present Absent/present Absent/present Absent/present
Mastoid temporal border		
Articulation facet	Square or round with smooth cortical bone Square or round with ridgelike metaphyseal surface	Absent/present Absent/present
Morphology	Rectangular extending into the intracranial and extracranial surface	Absent/present

Refer to Fig. 1 for general anatomy and borders of the pars lateralis

## Data analysis

### Bone mineral density patterning

The bone mineral density patterning was assessed by means of calculating a bone mineral density ratio at each region of interest across the left pars lateralis. To calculate the BMD ratio, the maximum grey value assessed at each region of interest point was used relative to the maximum grey value of the reference material included in each scan, i.e. the maximum grey value of each assessment point was subdivided by the maximum grey value of the reference material. This resulted in the data for each assessment point being presented as a ratio, which allowed for further statistical analysis and comparisons of the bone mineral density distribution values across the sample. Each BMD ratio was thus used to show the pattern of change in BMD relative to other BMD sites across the pars lateralis and as such served as a reflection of potential ossification of the bone. Descriptive statistics were applied to assess bone mineral density ratios associated with skeletal sites using GraphPad Prism version 3.4.1 (GraphPad Software Inc., San Diego, California, USA).

### Geometric morphometrics

A Generalised Procrustes Superimposition, which compared the relative position of each landmark whilst accounting for rotation and scaling, was used to study shape changes across the sample. Landmark data was processed using Morphologika version 2.5 (University of York, United Kingdom) [31]. Wireframes of the mean shape for each age group were used to visualise the intracranial, lateral, and hypoglossal view. Principal component analysis (PCA) was utilised to assess covariation of variables across a sample [30] and was run using Paleontological Statistics Software (PAST) version 4.03 [32].

Size data was analysed using SPSS version 22 (Statistical Package for the Social Sciences, IBM Corporation., 2013). A Shapiro–Wilk test was run for each measurement (Fig. 3) to assess the distribution of the sample. As the sample was normally distributed, the appropriate means and standard deviations were calculated. A multivariate analysis of variance (MANOVA), with a Tukey-HSD post hoc analysis was then used to assess the relationship between the age category relative to each measurement, where *p* ≤ 0.05 was considered statistically significant.

### Stereomicroscopy

Chi-square tests of independence were performed to examine the relationship of osteomorphology and biological age using SPSS version 22 (Statistical Package for the Social Sciences, IBM Corporation., 2013). Frequency distributions were recorded to assess the presence of specific morphology associated with biological age.

To account for inter and intraobserver error, a sample of 11 pars laterali across the age groups was randomly selected and subjected to each of the assessed techniques. A Lin’s concordance correlation was applied to the measurements where a value between 0.81 and 1.00 indicated a high level of repeatability [33]. Inter and intraobserver repeatability ranged between 78 and 97% for all measurements. Reproducibility in plotting and determining the bone mineral density ratios at the specific sites of the pars lateralis was validated by an interobserver error range of 81–99.4%.

## Results

### Bone mineral density patterning

**Fig. 4:**
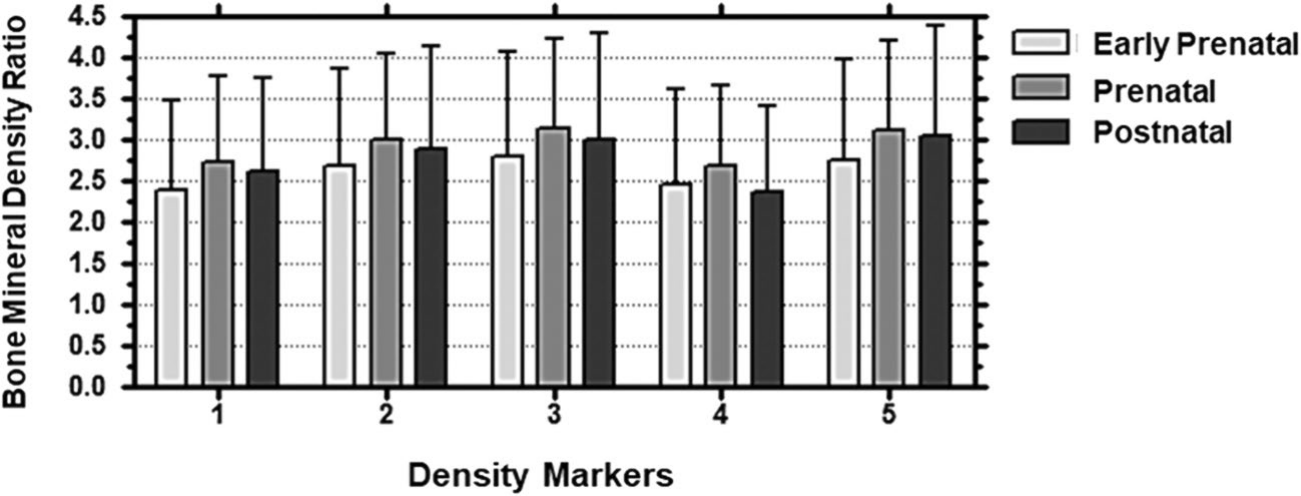
Two-way ANOVA. Bone mineral density ratio means and standard deviation within age groups for the pars lateralis. < 30 gestational weeks—early prenatal (*N* = 19), 30–40 gestational weeks—prenatal (*N* = 20), birth–7.5 months—postnatal (*N* = 22). Density markers: (1) jugular limb facet, (2) condylar limb facet, (3) inferior border of the foramen magnum, (4) medial edge of the posterior region for the supraoccipital (pars squama) of the occipital complex, (5) primary ossification site. Analysis by GraphPad 4 Software

Bone mineral density (BMD) points in prenatal individuals were higher when compared to those in the early prenatal age group across all BMD points examined. Density mean ratios were larger in the prenatal group when compared with those in the early prenatal group for the five points examined. BMD in postnatal individuals was lower when compared with that in the prenatal individuals. No statistically significant differences between points were noted (Fig. 4).

### Geometric morphometrics

The maximum width and maximum length of the pars lateralis were significantly larger in the postnatal age group when compared to the prenatal age group (*p* < 0.001). In addition, the maximum width and maximum length of the pars lateralis were significantly larger in the prenatal group when compared to the early prenatal group (*p* < 0.001)(Table 4).

**Table 4:** Means and standard deviations of the measurements assessed on the pars lateralis

	Early prenatal (20–30 gestation weeks)	Prenatal (30–40 gestational weeks)	Postnatal (birth–7.5 months)	Tukey-HSD post hoc	
	*n* = 32	*n* = 40	*n* = 30	Early prenatal vs prenatal	Prenatal vs postnatal
Maximum width	6.88 ± 0.24	9.73 ± 0.32	13.73 ± 0.38	*p* < 0.001	*p* < 0.001
Maximum length	12.21 ± 0.44	16.86 ± 0.57	22.74 ± 0.53	*p* < 0.001	*p* < 0.001

Measurements expressed as mean ± SD. Data was normally distributed

**Fig. 5:**
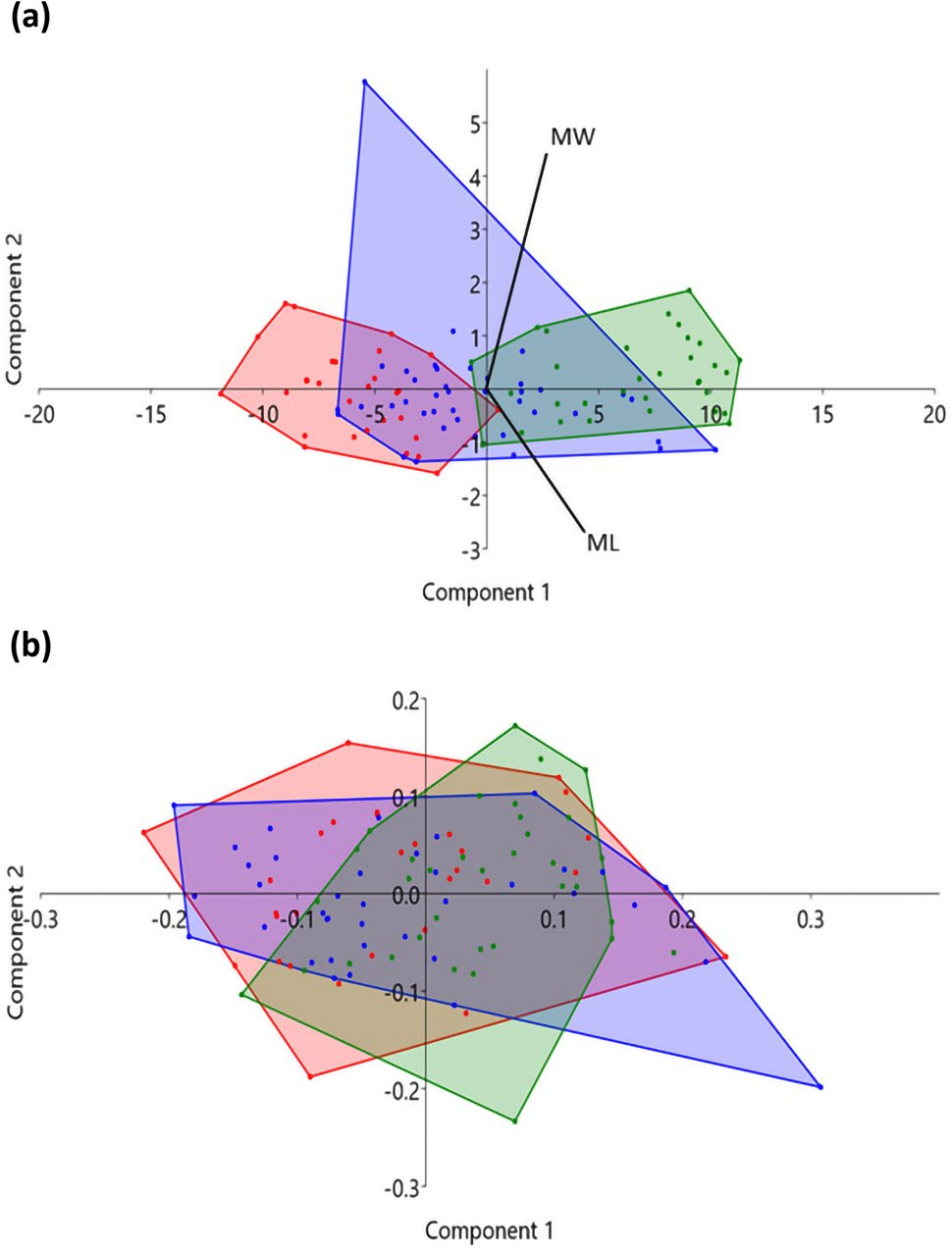
Principal component analysis (PCA) assessing the degree of variance in the size of the pars lateralis between early prenatal (red, less than 30 gestational weeks), prenatal (blue, 30 gestational weeks–birth) and postnatal (green, birth–7.5 months postnatal) age categories. Component 1 represents size and shape. Component 2 represents size. Biplots illustrate the influence of each measurement on PC1 and PC2. In the PCA, an overlap of the group distribution map (coloured areas) indicates little to no variation between the represented groups, whilst a separation between groups indicates a high to moderate degree of variation between the represented groups. **a** Intracranial view. **b** Hypoglossal region

In the PCA of the pars lateralis, a high degree of overlap was observed between the early prenatal and prenatal groups as well as between the prenatal and postnatal age groups (Fig. 5a). A high degree of overlap between all three age groups was observed in the hypoglossal region (Fig. 5b). The high degree of overlap between groups is indicative of a lower degree of variation across the sample between age groups.

**Fig. 6:**
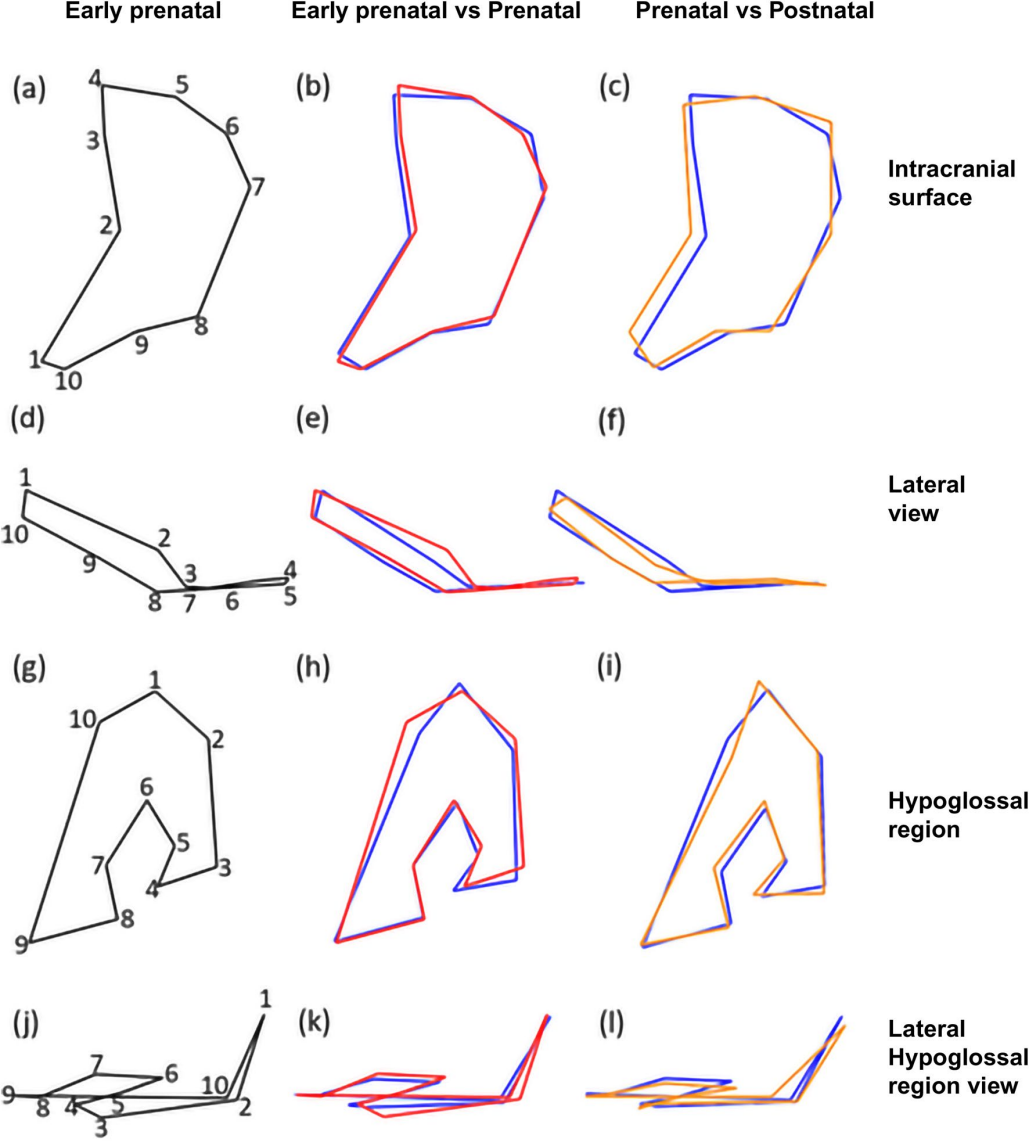
Wireframes illustrating the intracranial and lateral shape of the pars lateralis and hypoglossal region. Intracranial surface. **a** Early prenatal stage: less than 30 gestational weeks. **b** Prenatal (blue) intracranial surface wireframe superimposed on early prenatal (red) intracranial surface wireframe. **c** Postnatal (orange) intracranial surface wireframe superimposed on prenatal (blue) intracranial surface wireframe. Lateral view. **d** Early prenatal stage. **e** Prenatal (blue) lateral view wireframe superimposed on early prenatal (red) lateral view wireframe. **f** Postnatal (orange) lateral view wireframe superimposed on prenatal (blue) lateral view wireframe. Hypoglossal region. **g** Early prenatal stage. **h** Prenatal (blue) hypoglossal region wireframe superimposed on early prenatal (red) hypoglossal region wireframe. **i** Postnatal (orange) hypoglossal region wireframe superimposed on prenatal (blue) hypoglossal region wireframe. Lateral hypoglossal region view. **j** Early prenatal stage. **k** Prenatal (blue) lateral hypoglossal region view wireframe superimposed on early prenatal (red) lateral hypoglossal region view wireframe. **l** Postnatal (orange) lateral hypoglossal region view wireframe superimposed on prenatal (blue) lateral hypoglossal region view wireframe. Numbers 1–10 indicate landmarks

When visualising shape further using the wireframes depicting the intracranial view of pars lateralis (Fig. 6a–c), the distances between posteromedial corner (landmark 4) and the landmark on the mid posterior border (landmark 6) as well as between the posteromedial corner (landmark 4) and the posterior lateral facet (landmark 7) were wider in postnatal individuals when compared to the younger age cohorts. The lateral facets (landmarks 7 to 8) appeared to be shorter and more oblique in the postnatal individuals when compared to the prenatal individuals (Fig. 6c). The concavity of the anteromedial angle created across landmarks 1 to 4 appeared more obtuse in the postnatal group when compared to the prenatal group (Fig. 6c). The angle at the anterolateral corner (between landmarks 8–9-10) appeared deeper and more defined in the postnatal group when compared to the prenatal and early prenatal groups, respectively. The deeper angle created between landmarks 8 and 10 was coupled with a reduction in the distance between the anteromedial angle (landmark 2) and the border of the jugular foramen (landmark 9) in the postnatal group when compared to the younger age cohorts. In the lateral view, the jugular limb (landmarks 1 and 10) appeared shorter relative to its height with each subsequent age category (Fig. 6d–f).

In the hypoglossal region, the curve created between the superior jugular limb (landmark 3), inferior jugular limb (landmark 4) and the landmark at the jugular mid anteromedial angle (landmark 5) (Fig. 6g) appeared more acute in the postnatal group when compared to the younger cohorts (Fig. 6h, i). The distance between the inferior jugular limb (landmark 10) and the furthest point on the jugular limb (landmark 2) was shorter in the prenatal group when compared to the early prenatal group (Fig. 6h). In the lateral view (Fig. 6j–l), the jugular tubercle (between landmarks 2 and 3) appeared to be more oblique in the early prenatal group when compared to the prenatal group (Fig. 6k) as well as when comparing the prenatal and postnatal age groups, respectively (Fig. 6l). Whilst in the postnatal age group the anteromedial angle point (landmark 6) appeared level with the condylar (landmarks 8 and 9) and jugular tubercles (landmarks 3 and 4) (Fig. 6l), this landmark was located more superiorly in the prenatal group. In the prenatal age group, the distance between the inferior jugular limb (landmark 10) and the jugular tubercle (landmark 2) appeared wider when compared to the postnatal age cohort (Fig. 6l). The inferior jugular limb (landmark 10) and the jugular tubercle (landmark 2) were oriented perpendicular to the posterior corner (landmark 1) in the early prenatal and prenatal age groups (Fig. 6k) which changed to a more outward extended appearance in the postnatal group (Fig. 6l).

### Stereomicroscopy

**Fig. 7:**
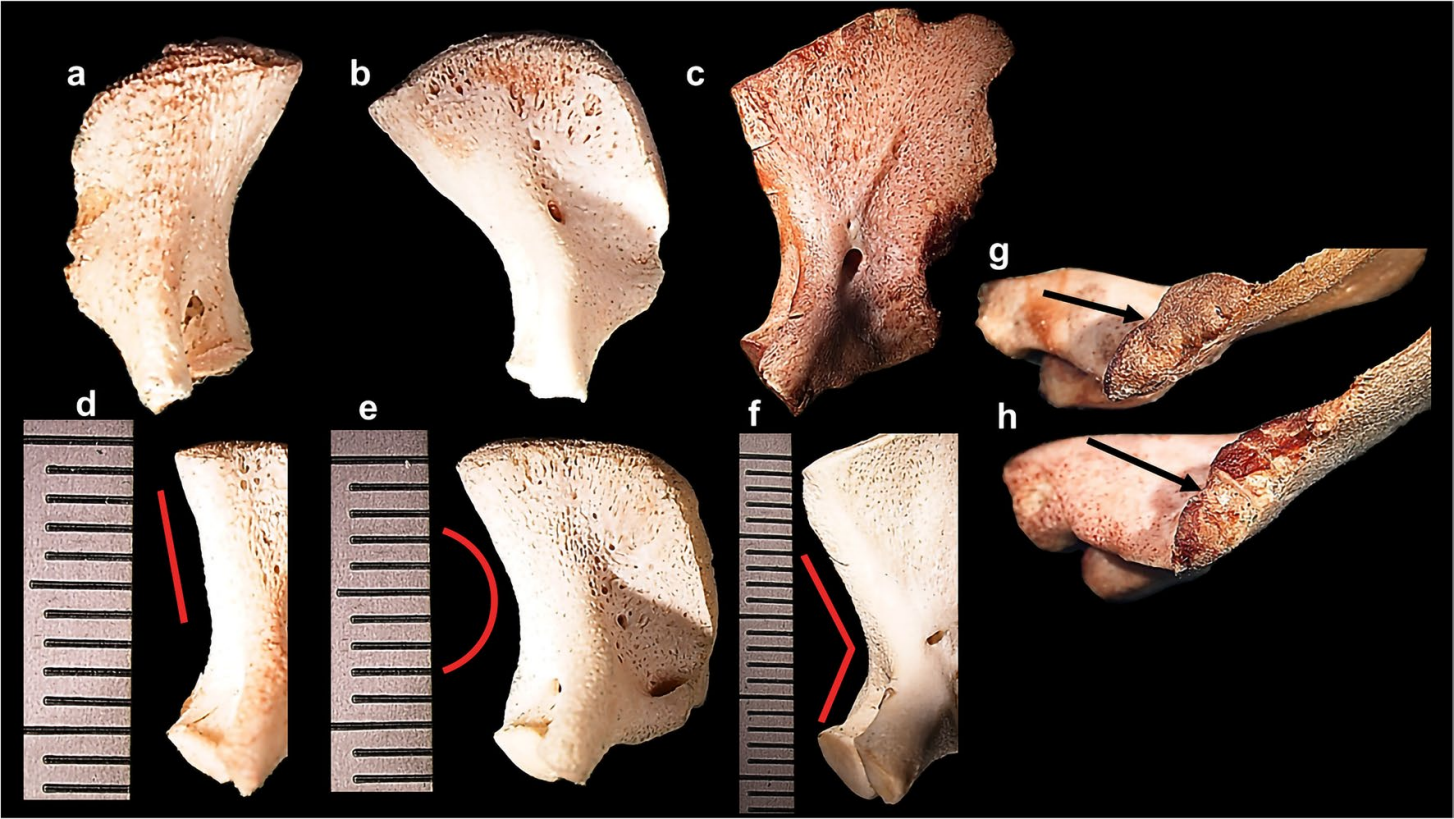
Pars lateralis bone shape and border morphology. **a** Magnified intracranial view of triangular early prenatal (< 30 gestational weeks) right pars lateralis element; scale, 9 mm. **b** Magnified intracranial view of quadrilateral prenatal (34–38 gestational weeks) left pars lateralis element; scale, 13 mm. **c** Magnified intracranial view of fan-like quadrilateral postnatal (birth–1.5 months) left pars lateralis element; scale, 23 mm. **d** Intracranial view of early prenatal (< 30 gestational weeks) left pars lateralis element exhibiting thickened straight medial border for the foramen magnum (red line); scale, 12 mm. **e** Intracranial view of prenatal (34–38 gestational weeks) left pars lateralis element exhibiting thickened rounded medial border for the foramen magnum (red line); scale, 12 mm. **f** Magnified view of intracranial surface of postnatal (birth–1.5 months) left pars lateralis element exhibited thickened V-shaped medial border for the foramen magnum (red line); scale, 22 mm. **g** Magnified mastoid temporal facet of prenatal (34–38 gestational weeks) left pars lateralis element exhibiting smooth metaphyseal surface (black arrow). **h** Magnified mastoid temporal facet of postnatal (birth–1.5 months) exhibiting ridgelike metaphyseal surface (black arrow). Magnification (1.5–2.5 ×) performed on Nikon Stereomicroscope (SMZ1500, Japan)

The pars lateralis appeared as a quadrilateral plate in 50 bones included in the sample (*n* = 50/99) (Table 5). The overall bone shape for early prenatal elements was triangular (*n* = 19/29; 65%) (Fig. 7a), followed by quadrilateral in the prenatal elements (*n* = 33/40; 82%) (Fig. 7b) and a fan-like quadrilateral element (*n* = 23/30; 76%) in the postnatal stage (Fig. 7c). The medial border of the pars lateralis, forming the medial edge of the foramen magnum, was straight in the early prenatal group (*n* = 19/29; 65%) (Fig. 7d), curved and concave in the prenatal group (*n* = 23/40; 57%) (Fig. 7e) and V-shaped in the postnatal group (*n* = 22/30; 73%) (Fig. 7f). A higher incidence of the articular facet located on the mastoid temporal border was observed in the postnatal age group (*n* = 28/39) when compared to the prenatal (*n* = 10/39) and early prenatal (*n* = 1/39) age groups, respectively. Typically rectangular in shape, the borders of the mastoid temporal border articular facet extend towards the extracranial and intracranial surfaces in the postnatal age group (*n* = 24/29; 83%) (Fig. 7g, h). The metaphyseal surface comprised of smooth cortical bone (*n* = 29/97; 30%) in the prenatal and postnatal age groups (Fig. 7g). A striated or ridgelike morphology was observed in 11 skeletal elements from the postnatal cohort (Fig. 7h).

**Table 5:** Frequency of morphological features of the left pars lateralis bone shape and border morphology

Morphological feature	Age category (*N*)			Total (*N*)
	Early prenatal (30)	Prenatal (41)	Postnatal (30)	101
Bone shape	**29**	**40**	**30**	**99**
Triangular	19	/	/	19
Quadrilateral plate	10	33	7	50
Fan-like quadrilateral plate	/	7	23	30
Medial border of the pars lateralis	**30**	**40**	**30**	**100**
Straight and thin	1	/	/	1
Straight and thickened	19	12	2	33
Rounded and thickened	9	23	6	38
V-shaped and thickened	/	5	22	27
Mastoid temporal border	**29**	**39**	**29**	**97**
Articular facet present	1	10	28	39
Rectangular shape extending to the intracranial and extracranial surface	1	7	24	32
Square and smooth articular facet	3	11	15	29
Square and ridgelike articular facet	/	/	11	11

**Fig. 8:**
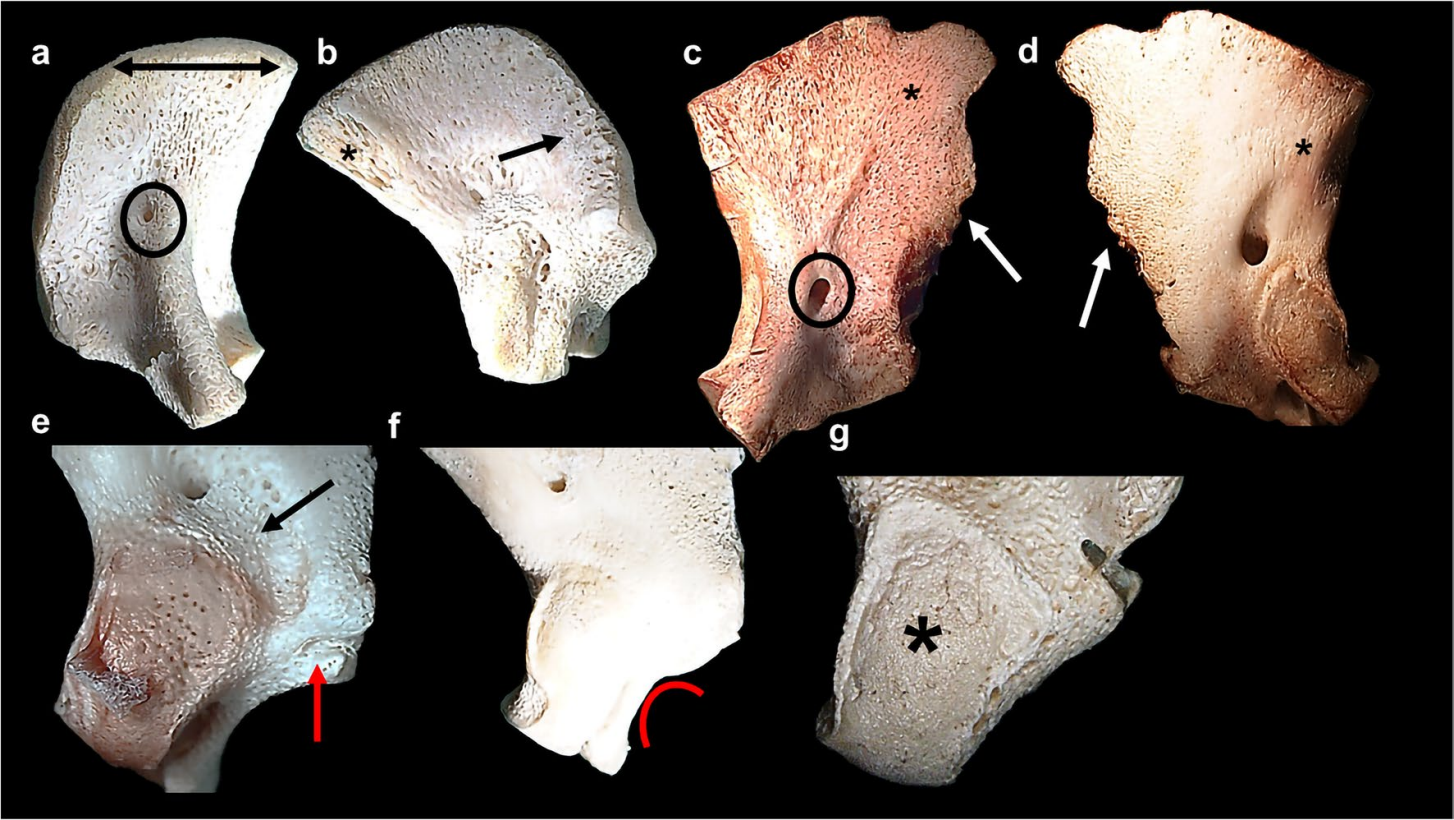
Pars lateralis intracranial and extracranial surface morphology. **a** Magnified intracranial view of prenatal (30–34 gestational weeks) right pars lateralis element exhibiting uniform ossification, posterior condylar canal (black circle) and 90° posterior border (double black arrow). **b** Magnified extracranial view of prenatal (30–34 gestational weeks) right pars lateralis element exhibiting uniform ossification, porous appearance (black arrow) and trabeculae(*). **c** Magnified intracranial view of postnatal (birth–1.5 months) left pars lateralis element exhibiting differential ossification, nutrient foramina (*), posterior condylar canal (black circle) and serrated mastoid temporal border (white arrow). **d** Magnified extracranial view of postnatal (birth–1.5 months) left pars lateralis element exhibiting differential ossification, longitudinal striations at the medial border for the foramen magnum (*) and serrated mastoid temporal border (white arrow). **e** Magnified extracranial view of prenatal (38 gestational weeks–birth) left pars lateralis element exhibiting immature jugular process (red arrow) and raised occipital condyle with billowing (black arrow). **f** Magnified extracranial view of postnatal (birth–1.5 months) left pars lateralis element exhibited developing border for the jugular foramen (red curved line). **g** Magnified extracranial view of postnatal (birth–1.5 months) left pars lateralis element exhibiting ridgelike occipital condyle (*). Magnification (1.5–2.5 ×) performed on Nikon Stereomicroscope (SMZ1500, Japan)

A uniform ossification patterning (*n* = 50/99; 50%) on the intracranial surface of the pars lateralis was consistently observed across all age groups (Table 6; Fig. 8a). The number of observations decreased in the postnatal age range (*n* = 8/50;16%) when compared with the early prenatal and prenatal age groups (21/50; 42%). The frequency of differential ossification patterning (*n* = 48/99) was more prevalent in the prenatal and postnatal age groups (*n* = 8/48; 17% early prenatal, *n* = 18/48; 37% prenatal and *n* = 22/48; 46% postnatal) (Fig. 8c). Trabeculae (*n* = 79/99; 79%) were observed at the supraoccipital and mastoid borders within the prenatal (*n* = 34/40; 85%) and postnatal (*n* = 26/30; 86%) age ranges. The porous appearance of the intracranial surface (*n* = 52/99; 52%) was prevalent in the postnatal age range (*n* = 25/30; 83%), specifically near the mastoid temporal and posterior border for the supraoccipital in postnatal elements (Table 6; Fig. 8c). The border for the supraoccipital presented with a 90° angle in 58% of individuals (*n* = 58/99) and was prevalent during the prenatal age range (*n* = 33/40; 82%) (Table 6; Fig. 8a). The mastoid temporal border was serrated in appearance (*n* = 34/99; 34%) in the postnatal cohort (*n* = 26/30; 87%) (Table 6; Fig. 8c). The presence of the posterior condylar canal was consistent across the age groups. However, variation was observed in terms of the location, size, and symmetry on the respective intracranial and extracranial surfaces (Fig. 8a, c).

**Table 6:** Frequency of morphological features of the left pars lateralis intracranial and extracranial surfaces

Morphological feature	Age category (*N*)			Total (*N*)
	Early prenatal (30)	Prenatal (41)	Postnatal (30)	101
Intracranial surface	**29**	**40**	**30**	**99**
Presence of posterior condylar canal	15	32	25	72
Uniform ossification pattern	21	21	8	50
Differential ossification pattern	8	18	22	48
Porous appearance	6	21	25	52
Trabecular appearance at borders	19	34	26	79
Nutrient foramen over surface	17	24	15	56
90-degree straightened posterior border	13	33	12	58
Serrated mastoid temporal border	/	8	26	34
Extracranial surface	**29**	**40**	**29**	**98**
Presence of posterior condylar canal	17	23	18	58
Uniform ossification pattern	19	27	13	59
Differential ossification pattern	10	12	16	38
Porous appearance	3	13	9	25
Longitudinal striated ossification pattern at borders	14	35	23	72
Nutrient foramen over surface	13	15	14	42
Presence of jugular process	4	20	24	48
90-degree straightened posterior border	14	36	15	65
Serrated mastoid temporal border	/	11	25	36
Rounded border for jugular foramen	10	20	27	57
Round and smooth occipital condyle	28	18	10	56
Raised and ridgelike occipital condyle	1	21	20	42
Billowing superior border of occipital condyle	23	24	24	71

Differential ossification patterning was observed on the extracranial surface (*n* = 38/98; 39%). Longitudinal striations (*n* = 72/98; 73%) were prevalent at the medial border of the foramen magnum in the prenatal (*n* = 35/72; 49%) (Fig. 8b) and postnatal (*n* = 23/72; 32%) (Fig. 8d) age groups, respectively (Table 6). A higher frequency in appearance of the immature jugular process (*n* = 48/98; 49%) was observed in the prenatal (*n* = 20/48; 42%) and postnatal (*n* = 24/48; 50%) age groups when compared with the early prenatal age group (*n* = 4/48; 8%) (Fig. 8e). In addition, the rounding of the border for the jugular foramen (*n* = 57) was observed in 90% of postnatal individuals (*n* = 27/30) (Table 6; Fig. 8f). The occipital condyle was observed as a round and smooth feature (*n* = 56/98; 57%) across the early prenatal (*n* = 28/56; 50%), prenatal (*n* = 18/56; 32%) and postnatal (*n* = 10/56; 18%) age ranges, respectively (Table 6; Fig. 8f). The occipital condyle appeared as raised and ridgelike (*n* = 42/98) in the prenatal (*n* = 21/42; 50%) and postnatal age ranges (*n* = 20/42; 48%), respectively (Fig. 8g).

**Fig. 9:**
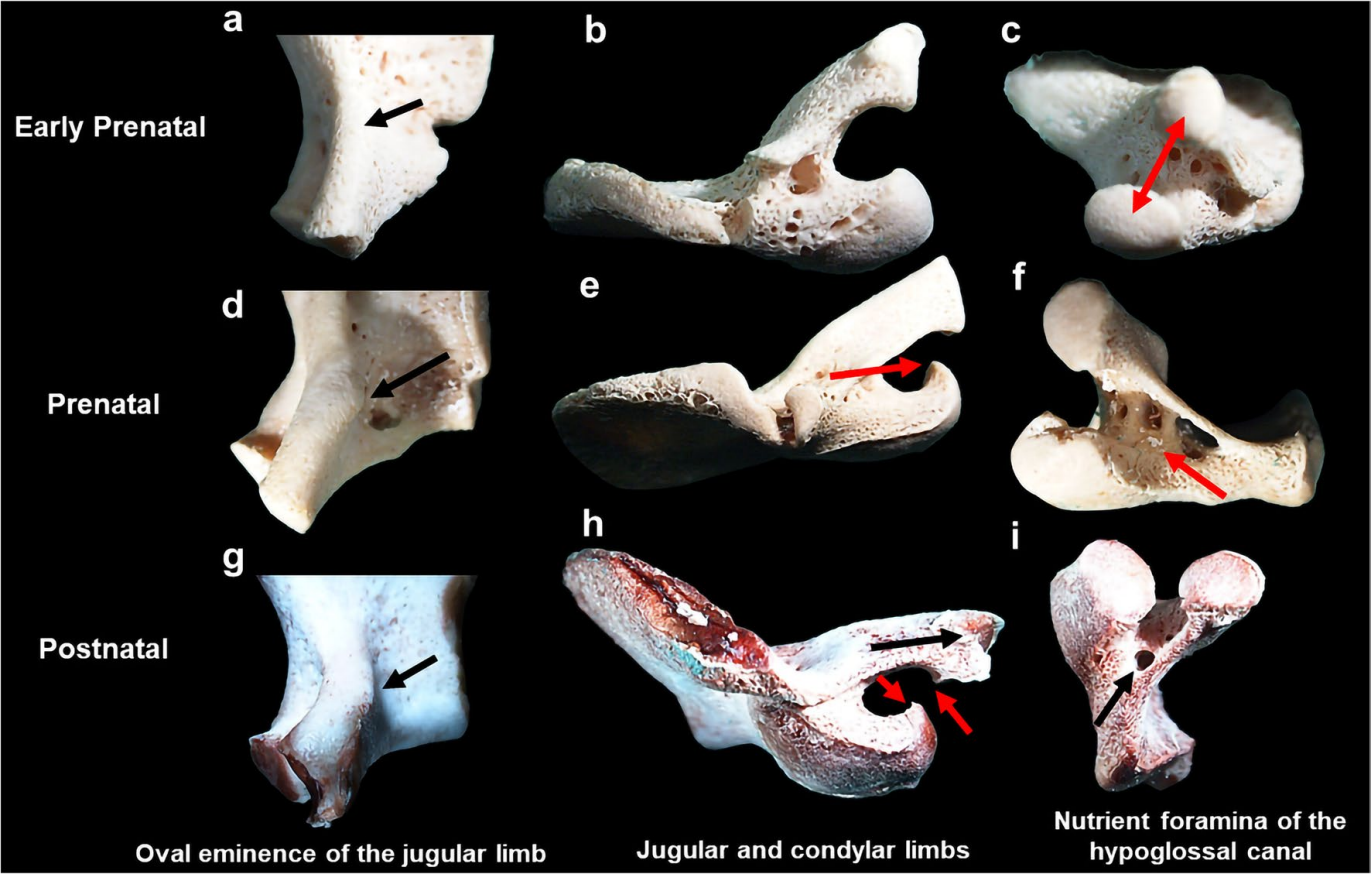
Pars lateralis hypoglossal region: magnified view of early prenatal (< 30 gestational weeks) left pars lateralis element (**a**–**c**). **a** Intracranial surface presenting straightened thick jugular limb with no jugular tubercle present. **b** Mastoid temporal region presenting straightened and thick jugular and condylar limbs. **c** Inferior view of articulation facets, round, and smooth cortical ossification of articular facets of the jugular and condylar limb (double red arrow). Magnified view of prenatal (38 gestational weeks–birth) left pars lateralis element (**d**–**f**). **d** Intracranial surface exhibiting developing oval eminence (black arrow). **e** Mastoid temporal region presenting with defined curve of shorter condylar limb (red arrow). **f** Inferior view of articular facets exhibiting vascular fovea at the intersect (red arrow). Magnified view of postnatal (1.5–4.5 months) left pars lateralis element (**g**–**i**). **g** Intracranial surface exhibiting prominent oval eminence of the jugular limb (black arrow). **h** Mastoid temporal region presenting pointed and lipping of the jugular articular facet (black arrow) and medially curved limbs with hooking of metaphyseal surfaces (red arrows). **i** Inferior view of articulation facets exhibiting vascular fovea at the intersection forming the immature hypoglossal canal (black arrow). Magnification (1.5–2.5 ×) performed on Nikon Stereomicroscope (SMZ1500, Japan)

The jugular limb appeared as a straight and thickened protuberance of bone, with an absent oval eminence and square articular facet comprising cortical bone in the early prenatal cohort (Fig. 9a–c, Table 7). The jugular limb presented with a distinctive oval eminence during the prenatal (*n* = 15/45; 33%) and postnatal (*n* = 30/45; 66%) stages of development (Table 7; Fig. 9d, g). The jugular limb presented as curved in prenatal (*n* = 7/34; 20%) and postnatal (*n* = 27/34; 79%) elements (Table 7; Fig. 9d). The articular facet of the jugular limb was found to be round and smooth across the age ranges examined (Fig. 9c, f, i). Metaphyseal surfaces of the articular facets presented with ridgelike morphology and lipping at the borders (*n* = 5/98; 5%) (Table 7; Fig. 9h) in the postnatal age group. Adjacent to the articular facet, the jugular limb appeared pointed (*n* = 42/98;43%) in the prenatal (*n* = 18/42; 43%) and postnatal (*n* = 24/42; 57%) age groups (Table 7; Fig. 9g, h).

**Table 7:** Frequency of morphological features of the left pars lateralis hypoglossal region

Morphological feature	Age category (*N*)			Total (*N*)
	Early prenatal (30)	Prenatal (41)	Postnatal (30)	101
Jugular limb	**29**	**40**	**30**	**99**
Oval eminence of jugular tubercle	/	15	30	45
Straight, thick protuberance from intracranial surface	29	33	3	65
Curved, thick protuberance from intracranial surface	/	7	27	34
Square or rounded at articular facet	29	40	30	99
Pointed at articular facet	/	18	24	42
Articular facet round and smooth	29	37	24	90
Articular facet round, ridgelike and lipping of borders	/	/	5	5
Condylar limb	**29**	**40**	**30**	**99**
Minor curvature towards the jugular limb	28	29	5	62
Defined curvature of thick protuberance from extracranial surface towards the jugular limb	/	10	25	35
Square or rounded at articular facet	29	40	30	99
Pointed at articular facet	/	27	28	55
Articular facet round and smooth	29	38	27	94
Articular facet round, ridgelike and lipping of borders	/	/	/	0
Hypoglossal canal	**29**	**40**	**30**	**99**
Curvature medially of jugular and condylar limbs	2	13	24	39
Condylar limb shorter than the jugular limb	4	12	16	32
Nutrient foramina and vascular fovea in intersection of limbs	28	35	26	89
Hooking of the articular facets medially	5	22	28	55

Similar features were observed across both the condylar and jugular limbs which included the round and square shape at the articular facet and smooth cortical bone on the metaphyseal surface (Table 7; Fig. 9c, f). A minor curvature of the condylar limb (*n* = 62/99; 63%) was present in the early prenatal (*n* = 28/62; 45%) and prenatal skeletal elements (*n* = 29/62; 47%) (Fig. 9b, e). The condylar limb curvature (*n* = 35/99; 35%) appeared more distinct in the postnatal age range (*n* = 25/35; 72%), when compared to the prenatal elements (*n* = 10/35; 28%) (Table 7; Fig. 9h). The ridgelike morphology with lipping observed on the jugular limb articular facet was absent on the condylar limb in the sample. The condylar limb articular facet presented with a pointed appearance (*n* = 55/99) in the prenatal (*n* = 27/55; 49%) and postnatal (*n* = 28/55; 51%) age groups, respectively (Fig. 9e, h).

A consistent finding was the presence of vascular fovea and/or nutrient foramina at the intersection of the jugular and condylar limb (Fig. 9c, f, i). These vascular foveae were more pronounced and defined in the prenatal age group (Fig. 9f). The medial curvature of jugular and condylar limbs which form the hypoglossal canal (*n* = 39/99; 39%) was more prevalent in the prenatal (*n* = 13/39; 33%) and postnatal (24/39; 61%) age groups when compared to only two observations in the early prenatal age group (Table 7). The condylar limb was found to be typically shorter than the jugular limb (*n* = 32/99; 32%), particularly in postnatal individuals (*n* = 16/32; 50%) (Table 7; Fig. 9e). The hooking of the articular facets medially (*n* = 55/99; 55%) (Fig. 9e, h) was found in majority of postnatal individuals (*n* = 28/30; 93%).

## Discussion

As a major contributor to the osteology of the immature posterior cranial fossa, a comprehensive understanding of the development of the pars lateralis is necessary when one considers its involvement in craniovertebral anomalies, skull base disorders and occipital fractures [34, 35]. Rapid development and growth of the morphological features of the immature skeleton aid in the accurate age estimation of and subsequent identification of human remains within a forensic setting [2, 3, 15, 16, 36, 37]. The morphology of the pars lateralis is understudied, partly due to the fragile nature of the pars lateralis mastoid temporal border, which remains cartilaginous until late prenatal life [27]. The current study was undertaken to generate morphological findings which distinguish the immature pars laterali from bones of similar morphology, i.e. the immature scapula [38]. In addition, the study aimed to provide a comprehensive account of changes in the osteology of the pars lateralis to assist forensic practitioners in their estimations of age using cranial base skeletal elements. Findings include statistically significant differences in size and shape between the early prenatal, prenatal, and postnatal age groups which accommodate neurovascular structures and skeletal elements. Stereomicroscopy and geometric morphometrics both highlighted the change in shape of the pars lateralis, as well as morphology which is associated with biological age, observed at the borders and sites of articulation.

The overall shape of the pars lateralis transitioned from a triangular shape in the early prenatal age group to quadrilateral in prenatal individuals and then finally fan-like quadrilateral bone in postnatal individuals. The angulation of the medial border for the foramen magnum observed between landmarks 1 and 4 highlighted a change in shape between straight in the early prenatal cohort to V-shaped in the postnatal individuals. In addition, the perpendicular posterior border for the pars squama, which was prevalent in the prenatal cohort, decreased in number of observations as the mastoid temporal border appeared serrated in the postnatal cohort. Furthermore, the distance between the posteromedial border (landmark 4) and the posterior lateral facet (landmark 7) increased in postnatal individuals when compared to the younger age cohorts. These observations indicate an expansion of ossified tissue towards the pars temporalis and supraoccipital portion of the complex. These illustrated shape changes may be dependent on the increase in maximum width and length across the sample which agrees with previous observations [2, 3, 15, 16, 36–38]. The geometric morphometric data generated validates measurement criterion previously demonstrated by dry bone anthropometrics as well as emphasises the shape changes of these cranial elements in the age ranges examined.

When further considering the expansion of the pars lateralis over the assessed period of growth it is important to consider the ossification patterning results which indicated the degree and direction of bone modelling during the prenatal and postnatal age ranges. The appearance of features associated with bone formation such as longitudinal striations near borders, trabeculae at borders, and a porous appearance over bone surfaces was observed at a high frequency in the prenatal cohort. In addition, evidence of vascularization was prevalent in the prenatal group due to the high frequency of vascular foveae present for the immature hypoglossal canal. All observations agree with similar observations made by Zdilla [21] who studied vascular patterns in the immature pars basilaris. The vascular model of ontogeny was supported in this work as the location of foveae and canals observed on the dorsal and ventral surface of the foetal pars basilaris was attributed to venous connections between the basilar plexus and pharyngeal venous plexus. The hypoglossal canal transmits the hypoglossal nerve, meningeal branch of the pharyngeal artery and emissary vein [39]. As such, the inferior region of the pars lateralis is closely associated with neurovascular structures. Recent advances on angiogenesis in bone physiology and regulation indicate that the role of vasculature is not reserved to oxygen and nutrient supply. Furthermore, bone angiogenesis is critical to regulating bone development and is closely related to osteogenesis and chondrogenesis [39, 40].

Of particular interest in the current study was the absence of statistically significant differences in the assessed bone mineral density across various regions of interest within the three age groupings examined. The absence of significant differences in bone mineral density coupled with the observed morphology, e.g. longitudinal striations, supports the hypothesis of the basicranial skeleton undergoing uniform development and growth across its entirety in the prenatal period in support of brain development [35, 41]. However, uniform bone mineral density of the pars lateralis observed in the current study disagrees with previous reports of changes in bone density across the embryonic murine skeleton [42], vertebral bone development [43, 44] and the immature mandible [25] when placed within the context of potential changes as a result of biomechanical influences associated with growth. A plausible reason for this relies on the age of individuals included as well as the regions of interest examined. Results from the current study suggest that the effect of biomechanical force and local soft tissue loading at this specific region of the cranial base is minimal at this stage of development. This is particularly relevant considering independent head movement and the developing atlas [45], which articulates with the condyles of the pars laterali. In addition, newly formed muscle, and contractions thereof, will modulate ossification rates [46]. Stereomicroscopy indicated that the occipital condyle presented as raised and ridgelike in 66% of the postnatal age group. This finding is indicative of development for articulation with the atlas. The bone quality of the occipital condyle was not included as a region of interest in the current study design. It is recommended that future imaging studies include assessment of the extracranial pars lateralis in older immature individuals (> 8 months postpartum) as a measure of evaluating biomechanical influence on paediatric head and neck anatomy. Overall, findings suggest that the form of the pars lateralis is of greater importance than mineralisation prior to 7 months postpartum. Ossification has been reported as more important to gestational age estimation as well as diagnostic of congenital disorders in foetuses younger than 30 gestational weeks, as ossification centres grow linearly with respect to sagittal and transverse diameters based on CT data [47]. The pars basilaris and pars laterali grow in tandem. However, the growth trajectory of the pars lateralis was found to be faster than the pars basilaris in two archaeological European samples [37]. Therefore, the overall architecture of the pars lateralis needs to be established prior to bone quality during the prenatal and postnatal stage of human cranial base development.

In contrast with the intracranial surface of the pars lateralis, maturation features for the extracranial region of the pars lateralis, specifically the jugular process and foramen, were recorded at a high frequency for postnatal elements. The distance between the anteromedial angle and the border of the jugular foramen (landmarks 2 and 9) was smaller in the prenatal and postnatal age groups when compared to the early prenatal age range. Therefore, indicating a narrowing of the bone as the border for the jugular foramen appeared more defined and deeper (landmarks 8–10) in the postnatal cohort. Stereomicroscopy data supports this finding as the rounding of the border for the foramen magnum and the immature jugular process was prevalent in postnatal individuals. These results highlight the importance of the jugular foramen as instrumental for the transmission and support of crucial neural and venous structures such as the jugular vein, glossopharyngeal, vagus and accessory nerves [48]. The rounding of the jugular foramen has been previously associated with bony bridge formation for articulation with the petrous part of the pars temporalis in foetal crania [49]. Bony bridge formation was not observed in the current sample, indicating a variable characteristic, which may be due to sociocultural differences [49]. Overall, findings agree with previous morphological summaries on the border of the jugular foramen and jugular process [10, 38], as the neonatal pars lateralis is characterised by the presence of these features.

In keeping with distinctive changes observed at the inferior aspect of the bone in the postnatal cohort, the morphology of the hypoglossal canal region appeared delayed when compared to the rest of the bone. PCA results indicated very little change between age stages, therefore suggesting that the shape and size of the hypoglossal region are not reliable for ageing of foetal decedents. The jugular and condylar limbs serve as branches for the intraoccipital anterior synchondrosis. Wireframe data represented changes across the cohort which are indicative of the hypoglossal canal developing to accommodate neurovascular structures [39]. However, these changes were minor and not discriminatory of a particular age range. Data on the anteromedial angle indicates that the jugular limb develops prior to the condylar limb. Observations included the presence of the oval eminence and medial curvature of the jugular limb and condylar limb in postnatal individuals. These findings are mirrored in the geometric morphometric data as the oval eminence was found to be oblique in the early prenatal cohort and an acute curve was observed with each subsequent age group (landmarks 10 and 2) as well as the shortening and definition of limb when compared to height (landmarks 1 and 10). This region has been found to be more reliable for age at death estimations during the postnatal stage of development following the first year of life, when considering the fusion of the hypoglossal canal and anterior intraoccipital synchondrosis with the pars basilaris [1, 11]. As such, the hypoglossal canal remains unfused during the periods examined in this study. It is proposed that future ageing criteria for the period of birth to 1 year of life would rely on changes observed at sites of articulation, in particular the specification of metaphyseal surface and hooking of the jugular and condylar limbs. However, these observations were found at a low frequency in the sample. As such, further enquiry is necessary to describe bone morphology prior to fusion.

Variables which influence the developing skeleton require attention when interpreting results from the current study. Social economic status including malnutrition, stress and poor resources impacts the developing skeleton [50–52]. Additionally, poor conditions will affect linear growth of the skeleton [53]. Consequently, the nature of the current sample should be noted where circumstances around potential concealment of birth and suspected infanticide may exist [27, 54, 55]. Inferences in the current study are limited by the absence of collateral information and data concerning maternal health and/or congenital abnormalities. Despite the limitations of the cross-sectional nature of the study, data represents specific growth phases which can be applied to forensic anthropology, and as such given the applicability and universal use of cranial anthropometric standards [37], morphological features and variants associated with traditional measurements have the potential to aid age at death estimations. This is particularly relevant to disaster victim identification, where a preliminary screening method prior to laboratory analyses would expedite identification. As such, the morphological findings generated from this sample require further quantitative testing and validation across archaeological and forensic contexts.

## Conclusion

The current investigation of the developing pars laterali has produced promising results in terms of shape differences and bone surface topography. These results support previous studies as well as contribute to the literature on immature bone morphology and forensic identification of subadult skeletal remains. The variation seen in the bone mineral density data indicates that the ossification and development of the pars laterali are stable which is in keeping with the premise of providing a stable anchor for the developing brain.

The various technical approaches used in the current study provided descriptive data and detail to fundamental information on the pars lateralis and thus advocates for further study into the reliability of morphological predictors on the surface of skeletal tissue as diagnostic criteria for biological profiling. Historically, morphological criterion and descriptions relating to the immature age groups are limited to ossification centre appearance [56], general shape [15] and epiphyseal union [57] in adolescent individuals. Prenatal and postnatal morphological summaries are limited by the prevalence of reference material [38], which impacts method development in the field of foetal osteology. Considering the high incidence of prenatal and postnatal deaths within the South African context, medicolegal investigation is challenged by the lack of standards and the conditions of remains admitted for medicolegal autopsy [54, 55]. This distinct setting allows for future advancements in forensic identification.

## Data Availability

Data is available upon reasonable request.
